# Gaze Self-Similarity Plot - A New Visualization Technique

**DOI:** 10.16910/jemr.10.5.3

**Published:** 2017-10-16

**Authors:** Pawel Kasprowski, Harezlak Katarzyna

**Affiliations:** Silesian University of Technology, Poland

**Keywords:** eye tracking, visualization, recurrence, visual patterns, classification

## Abstract

Eye tracking has become a valuable way for extending knowledge of human behavior based on visual patterns. One of the most important elements of such an analysis is the presentation of obtained results, which proves to be a challenging task. Traditional visualization techniques such as scan-paths or heat maps may reveal interesting information, nonetheless many useful features are still not visible, especially when temporal characteristics of eye movement is taken into account. This paper introduces a technique called gaze self-similarity plot (GSSP) that may be applied to visualize both spatial and temporal eye movement features on the single two-dimensional plot. The technique is an extension of the idea of recurrence plots, commonly used in time series analysis. The paper presents the basic concepts of the proposed approach (two types of GSSP) complemented with some examples of what kind of information may be disclosed and finally showing areas of the GSSP possible applications.

## Introduction

There are many visualization techniques for eye movement presentation among which scan-paths and heat maps showing spatial positions of gazes in relation to a stimulus come to the fore. The most important feature of the said visualization approaches is that they are straightforward and understandable even for laymen; however these techniques are not well suited to present temporal information. Temporal eye movement features such as fixations durations, their order and recurrence or saccades durations are not visible on heat maps and are barely visible on scan-paths, thus they have to be presented by means of other methods.

There are attempts to enrich scan-paths [ 1 ] or heat maps [ 17 ], but the general problem is that it is impossible to present three properties (horizontal and vertical position together with time) on a single two-dimensional plot. Therefore, many spatio-temporal visualization techniques use complex 3D graphs or combine different information in the same picture. See [ 3 ] for a state-of-the-art in this area.

The idea discussed in this paper alleviates the aforementioned problems by presenting spatial information by relative distances between gazes instead of their absolute locations. Such an approach - which was initially presented in [ 4 ] and significantly extended in the current research - allows to reduce one dimension.

The concept is based on the recurrence plot technique, used in the time series analysis to reveal repeating patterns in data [ 5 ]. This method has already been utilized in eye tracking field by [ 6 ] for a series of fixations located on axes X and Y according to their occurrence order. If fixation i_th_ and fixation j_th_ are close to each other, a point (i, j) on the plot is black, and when the distance between the fixations is above a threshold, it is white. Based on recurrence plot, several measures describing eye movement patterns have been defined. There are also tools for building recurrence plots, among which VERP Explorer is a good example [ 2 ].

A pattern created by a recurrence plot as used in [ 6 ] depends on two parameters - a maximal distance between two fixations to treat them as similar (or recurrent) and an algorithm for the fixation detection. It may be easily shown that the algorithm, which more eagerly merges subsequent fixations may provide a completely different plot and different values of recurrence measures, introducing this way some ambiguity.

In this paper we propose a visualization technique that does not depend on the previously mentioned parameters, because: (1) its functioning is not based on fixations, but on raw gaze coordinates, and (2) it visualizes a distance between gazes as a continuous value instead of using only two values indicating whether the distance is above or below the threshold, as in the case of the method described above. The next section of the paper introduces the technique, whereas in subsequent parts we present a non exhaustive list of possible applications of the method referred to as the Gaze Self-Similarity Plot (GSSP).

## Method

Suppose that we have a sequence of n gaze recordings g(1)...g(n) where each recording g(i) is described as a point in 2-dimensional space: (g_x_ , g_y_ ) . The x and y values are coordinates of a gaze on a screen with a resolution (x_max_, y_max_) . The GSSP is a visualization of a matrix consisting of n*n points where each point encodes a distance between an i_th_ and an j_th_ gaze points.

The GSSP is defined by the following equation:

(1) $$\begin{array}{ccc} \; & \begin{array}{l} gssp\left(i,j\right)=\frac{\sqrt{{\left(g_{x}(i)-g_{x}(j)\right)}^{2}+{\left(g_{y}(i)-g_{y}(j)\right)}^{2}}}{N} \end{array} & \; \end{array}$$

where N is the normalization factor, which is defined as the maximal possible distance between two gaze points:

(2) $$\begin{array}{ccc} \; & N=\sqrt{x_{\max}^{2}+y_{\max}^{2}} & \; \end{array}$$

Every element of the matrix may contain a value in range of (0...1) where 0 is represented by a black point and 1 is shown as a white one on the corresponding plot. The brightness of a pixel on such a plot informs about the Euclidean distance between two points. Black color means that two gaze points are very close to each other and white color indicates that the points are far from each other. The size of a plot is practically not limited and depends on the number of registered gazes.

**Figure 1:**
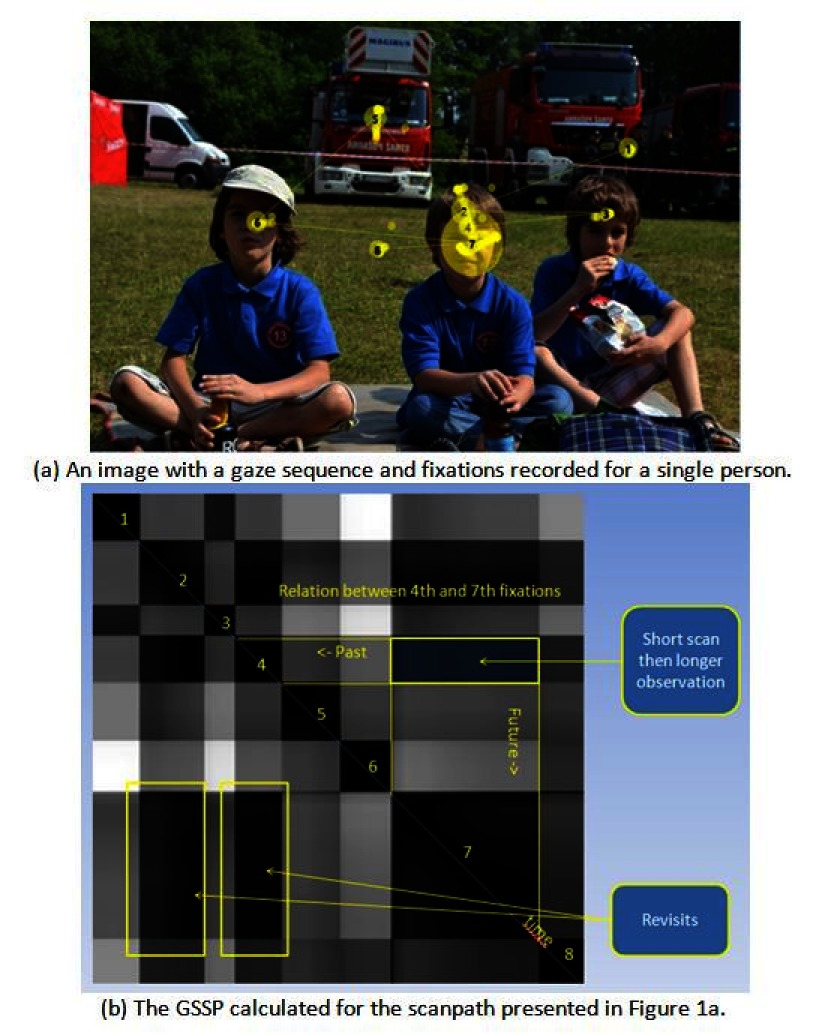
The GSSP example with the explanation of characteristic elements. Numbers from 1 to 8 denote black squares characteristic for fixations. On one hand, the GSSP shows that fixations 2, 4 and 7 appear very close to each other, which is a typical example of a recurrence behavior. On the other hand, fixations 1 and 3 are close to each other and very far from fixation number 6.

A sample recorded gaze sequence and the corresponding GSSP for that sequence with the description of its characteristic elements are presented in Figures 1a and 1b, respectively. The diagonal line from the upper-left corner (start) to the lower-right corner is black as it shows a distance of a gaze point to itself. Each group of black points adjacent to diagonal - visible as a black square - may be interpreted as a fixation. The bigger the square, the longer the fixation duration is. Rectangles outside the diagonal represent fixations distances. A dark rectangle indicates that two fixations are close to each other, which may be noticed in regard to fixations 2, 4 and 7 as well as to fixations 1 and 3. A bright rectangle indicates that groups of gaze points constituting fixations are far from each other, as in the case of fixations (1, 6) and (3, 6).

### Differentiating vertical and horizontal offsets using GSSP_VH_

The main disadvantage of recurrence plots, and at the same time of the GSSP presented above, is that the upper right part of the plot is a mirror of its lower left part. To avoid such a redundancy and to provide more information on the same plot we propose the extended version of the GSSP - denoted by GSSP_VH_ - in which the upper right part of the plot shows horizontal distances between gazes, while the lower left part presents vertical distances. Additionally, we propose to use the directed distances to preserve information not only about the distance, but also about the direction of the distance (e.g. from left to right or from right to left).

If we consider two gazes g (a) and g(b) for which a<b, i.e. g (a) was measured before g(b) , we can calculate horizontally and vertically directed distances as:

(3) $$\begin{array}{ccc} \; & \mathrm{d}x=\left(g_{x}(b)-g_{x}(a)\right)/x_{\max} & \; \end{array}$$

(4) $$\begin{array}{ccc} \; & \mathrm{d}y=\left(g_{y}(b)-g_{y}(a)\right)/y_{\max} & \; \end{array}$$

Therefore, the general formula for GSSP_VH_ calculation is:

(5) $$\begin{array}{ccc} \; & gssp_{vh}\left(i,j\right)=\left\{ \begin{array}{cc} -\mathrm{d}x, & i\geq j\\ \mathrm{d}y, & i<j \end{array}\right. & \; \end{array}$$

and every value may be in the range of (−1...1) .

It is worth noting that when condition i > j is fulfilled, it means that the gaze i was **after** the gaze j , so −dx must be taken as a directed distance.

Two ways to visualize such a matrix may be applied. One is to recalculate values to (0...1) range in the greyscale, similarly to the previous example. However, the main drawback of such an approach is that the distance equal to zero is difficult to distinguish visually, as after the recalculation it is equal to 0.5.

Therefore, we propose a colored plot and encoding each direction using a different color channel. For every point on the plot its color is defined using its three components (R, G, B): red (R), green (G) and blue (B). Every component may have a value in the range of (0...1) where 0 denotes lack of the component.

**Figure 2:**
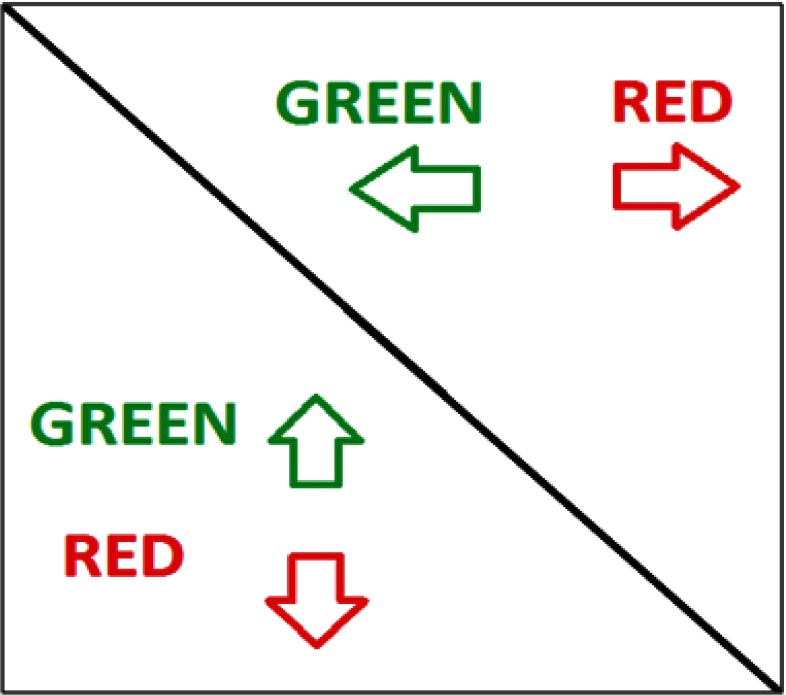
Illustration of the GSSP_VH_ idea. Horizontal distances are presented in the upper right part of the plot and vertical distances in the lower left one.

For instance, movements from left to right and from top to bottom may be characterized by a red component and from right to left and from bottom to top by a green component (but it is also possible to use any other color pattern) (see Figure 2).

For such color encoding every pixel value would be calculated as:

(6) $$\begin{array}{ccc} \; & I_{\left(R,G,B\right)}\left(i,j\right)=\left\{ \begin{array}{cc} I_{\left(gssp_{vh}(i,j),0 ,0\right)}, & gssp_{vh}\left(i,j\right)\geq 0\\ I_{\left(0,-gssp_{vh}(i,j),0\right)}, & gssp_{vh}\left(i,j\right)<0 \end{array}\right. & \; \end{array}$$

It is worth noting that a point may be only black, red or green and the intensity of a red or green component may change and it is not possible to have a point with both red and green components greater than 0.

**Figure 3:**
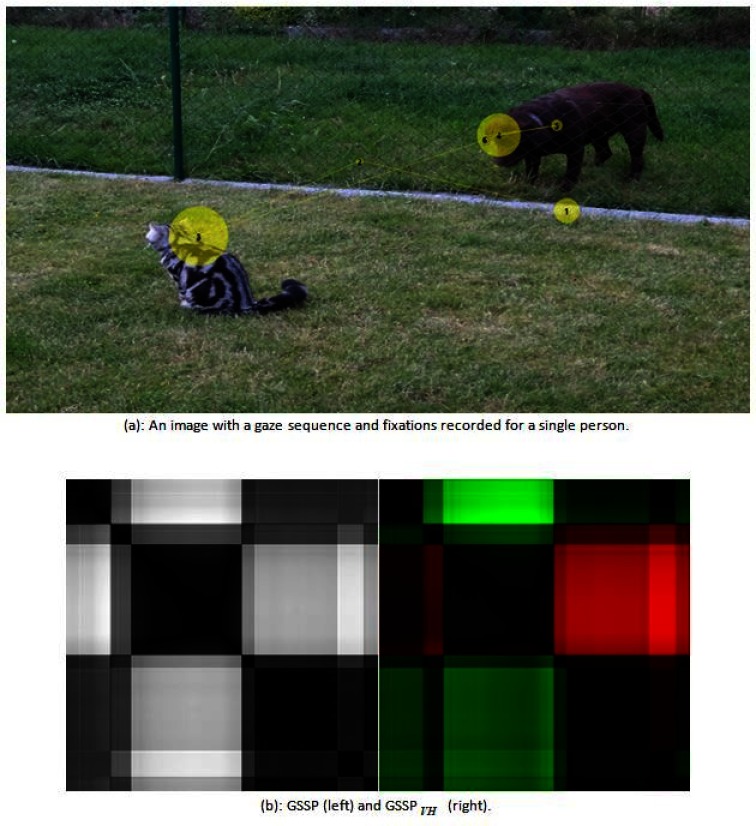
Figure 3b presents both GSSP and GSSP_VH_ plots for the gaze sequence shown in Figure 3a.

Figure 3b presents both types of GSSP calculated for the gaze sequence shown in Figure 3a.

An interesting property of GSSP_VH_ matrix is that it may be used to reconstruct a scan-path. The only required information is an absolute position of one gaze point. Having such a gaze point g_s_ (x_s_ , y_s_ ) we can calculate an absolute position of any other gaze point g_i_ (x_i_ , y_i_ ) using the following formulas:

(7) $$\begin{array}{ccc} \; & x_{i}=\left\{ \begin{array}{cc} x_{s}+gssp_{vh}\left(i,s\right), & i\geq s\\ x_{s}-gssp_{vh}\left(i,s\right), & i<s \end{array}\right. & \; \end{array}$$

(8) $$\begin{array}{ccc} \; & y_{i}=\left\{ \begin{array}{cc} y_{s}+gssp_{vh}\left(s,i\right), & i\geq s\\ y_{s}-gssp_{vh}\left(s,i\right), & i<s \end{array}\right. & \; \end{array}$$

### Quantitative Metrics for GSSP

Analysis of the above-described plots may reveal a lot of interesting information, which will be shown in further parts of the paper. However, comparison of several of such plots and their assessment based only on visual inspection may be difficult, thus we propose several quantitative metrics for GSSPs comparison. Since the GSSP is in fact an image, the metrics stem from image analysis algorithms.

The calculation of various characteristics of GSSP images has been based on the Co-occurrence Matrix (CM). It characterizes the texture of an image by determining how often pairs of pixels with specific values and in a specified spatial relationship occur in this image [ 7 ]. The size of CM is equal to the number of distinct values derived from the image, thus the calculation of CM must start with discretization of distances encoded in GSSP. All GSSP points must be recalculated from a continuous range of 0..1 to K discrete values forming a new matrix with integer values in range 0..K .

(9) $$\begin{array}{ccc} \; & I\left(x,y\right)=\left\lfloor gssp\left(x,y\right)*K\right\rfloor & \; \end{array}$$

where I(x,y) represents GSSP with recalculated values. Subsequently, CM (K + 1, K + 1) matrix is determined for every pair of values a = 0...K and b = 0...K and for a given offset d = (dx, dy) representing their spatial relationship. For the purpose of this research the value of K was arbitrarily set to 10.

In the case of GSSP_VH_ CM matrices are calculated separately for horizontal (upper right) and vertical (lower left) parts of the GSSP and are denoted by CM^V^ and CM^H^ respectively.

(10) $$\begin{array}{ccc} \; & cm_{\mathrm{d}x,\mathrm{d}y}^{H}\left(a,b\right)=\sum_{x=1}^{n-1}\sum_{y=x+1}^{n}\left\{ \begin{array}{cc} & I\left(x,y\right)=a\\ 1, & and\\ & I\left(x+\mathrm{d}x,y+\mathrm{d}y\right)=b\\ 0, & otherwise \end{array}\right. & \; \end{array}$$

(11) $$\begin{array}{ccc} \; & cm_{\mathrm{d}x,\mathrm{d}y}^{V}\left(a,b\right)=\sum_{y=1}^{n-1}\sum_{x=y+1}^{n}\left\{ \begin{array}{cc} & I\left(x,y\right)=a\\ 1, & and\\ & I\left(x+\mathrm{d}x,y+\mathrm{d}y\right)=b\\ 0, & otherwise \end{array}\right. & \; \end{array}$$

Co-occurrence matrices created in this way may serve to compute various image-related metrics.

### Homogeneity

The homogeneity of an image gives information to what extend nearby gazes are in similar locations. It is high when values in CM concentrate along the diagonal, meaning that there are a lot of pixels with the same or very similar value. The range of homogeneity is [0,1]. If an image is constant then homogeneity is equal to 1.

(12) $$\begin{array}{ccc} \; & homogeneity_{\mathrm{d}x,\mathrm{d}y}=\sum_{i=0}^{K}\sum_{j=0}^{K}\frac{cm_{\mathrm{d}x,\mathrm{d}y}\left(i,j\right)}{1+|i-j|} & \; \end{array}$$

### Contrast

The contrast is a difference moment of the CM and it measures the amount of local variations in an image. If the neighboring pixels have similar values then the contrast in the image is low. Therefore, the contrast is sensitive to long jumps from one gaze point to another. The range of contrast is [0, K ² ] where contrast is 0 for a constant image. The contrast is inversely proportional to homogeneity.

(13) $$\begin{array}{ccc} \; & contrast_{\mathrm{d}x,\mathrm{d}y}=\sum_{i=0}^{K}\sum_{j=0}^{K}{\left(i-j\right)}^{2}cm_{\mathrm{d}x,\mathrm{d}y}\left(i,j\right) & \; \end{array}$$

### Uniformity

Uniformity (also called energy) measures gaze pairs repetitions. It is high when the GSSP contains similar areas, which means that the same paired values with the same arrangement appear repeatedly in the image. It is low when there are no dominant pairs and the CM matrix contains a large number of small entries. The range of uniformity is [0,1], and it is 1 for a constant image.

(14) $$\begin{array}{ccc} \; & uniformity_{\mathrm{d}x,\mathrm{d}y}=\sum_{i=0}^{K}\sum_{j=0}^{K}{\left(cm_{\mathrm{d}x,\mathrm{d}y}(i,j)\right)}^{2} & \; \end{array}$$

For the purpose of the presented research all these metrics - homogeneity, contrast and uniformity - were evaluated taking into account the three offsets - vertical (0,1), horizontal (1,0) and diagonal (1,1).

## Experiments and Results

The usefulness of the GSSP was verified in terms of both visual exploration of registered eye movements and their quantification with the usage of the aforementioned metrics. In the first case the GSSP may prove useful in a quick identification of problems or in revealing characteristics of an eye movement patterns, not easily obtainable in case of a scan-path or heat map.

### Outlier detection

**Figure 4:**
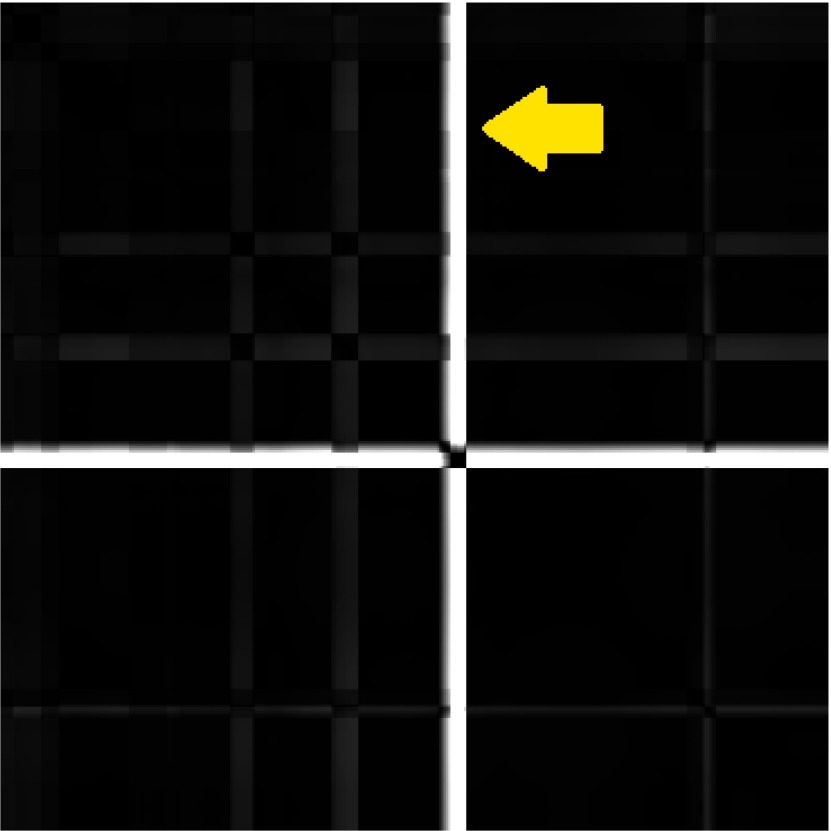
An example of the GSSP with visible outliers. The white cross with black square on the diagonal reveals several gaze points that are situated far away from all other points and may be treated as outliers. The three darker crosses show other possible outliers.

Outliers are visible on the GSSP plot as a bright cross with black square on a diagonal. One look at the GSSP gives information about the overall signal quality. Figure 4 presents the plot with one obvious outlier in the center of the plot and three more possible outliers. Of course evident outliers may be removed by means of simple analytic methods based on velocity thresholds [ 8 ], however the GSSP may be useful for examining the remaining scan-path to check for some less obvious outliers.

### Distinguishing regions of interest

**Figure 5:**
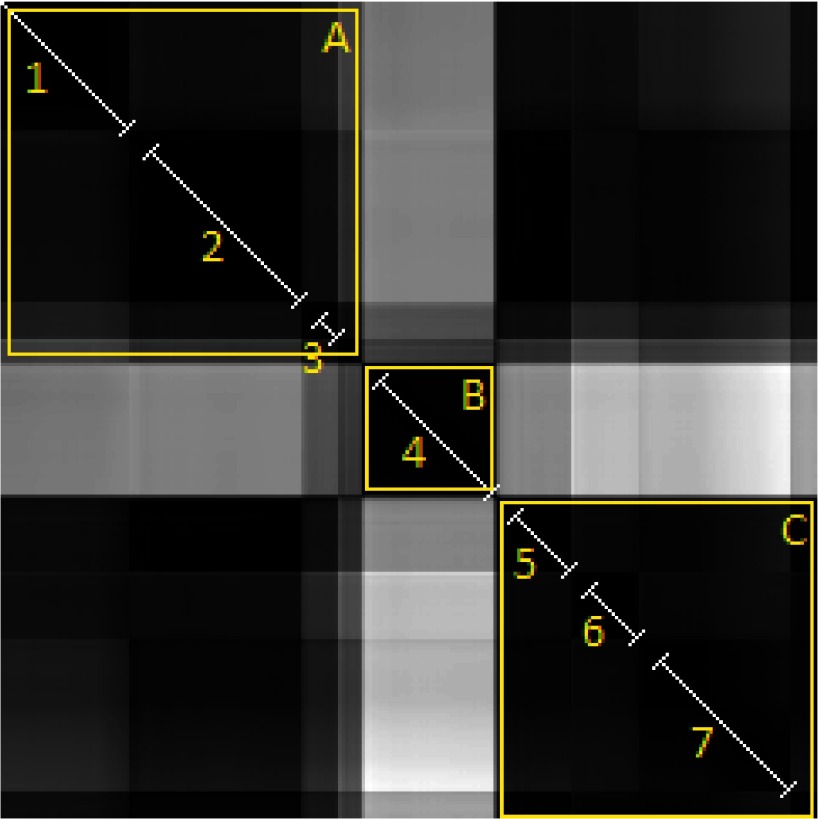
The GSSP with fixations (detected in a signal by the IDT algorithm) shown on diagonal as white lines. It is visible that despite seven fixations detected, there are three dark squares easily distinguishable along the diagonal, indicating three regions of interest (A, B and C). Additionally, dark rectangles out of diagonal show that the regions A and C are very close to each other, so they represent the same area of interest. It means that the observer looked at the first region (A), subsequently looked at the other (B) and then returned to the first one as region C is located in the same place as region A

Eye movement analysis is usually based on a fixations-saccades sequence extracted from a registered signal. It has been shown that such a sequence structure is sensitive to the fixation detection algorithm settings ( [ 9, 10 ]), and it is difficult to visually check, if the settings used are adequate. It became possible to present the detailed characteristics of fixations and saccades in 2D space on a single plot by means of the GSSP. We applied such a plot to estimate how homogeneous fixations are. On one hand, it may reveal that gaze points constituting the fixation are scattered, if there are shades on a fixation’s square. On the other hand, if two subsequent fixations appear in a similar place, they are visible as one big square. This gives the opportunity to observe a scan-path on a higher level - based on regions of interest instead of on separated fixations. An example is presented in Figure 5 on a plot with seven fixations and only two regions of interest.

### Recurrence of fixations

One of the main aims of the recurrence plots usage is revealing repeated pattern existing in time series. In the presented solution this feature was applied for the purpose of a registered gaze points analysis. Figure 5 presents the GSSP with a recurrence of gaze points’ placements. They are represented by dark rectangles appearing out of the diagonal line, which means that two groups of gaze points are located in the same place. In contrast to a classic recurrence plot, the proposed approach reveals not only repeated gaze points positions, but also allows - due to the application of the coloring mechanism - to estimate relative positions of the remaining points set. Additionally, the applied strategy of gazes’ presentation makes the simultaneous comparison of recurring fixations durations possible.

### Smooth pursuits visualization

**Figure 6:**
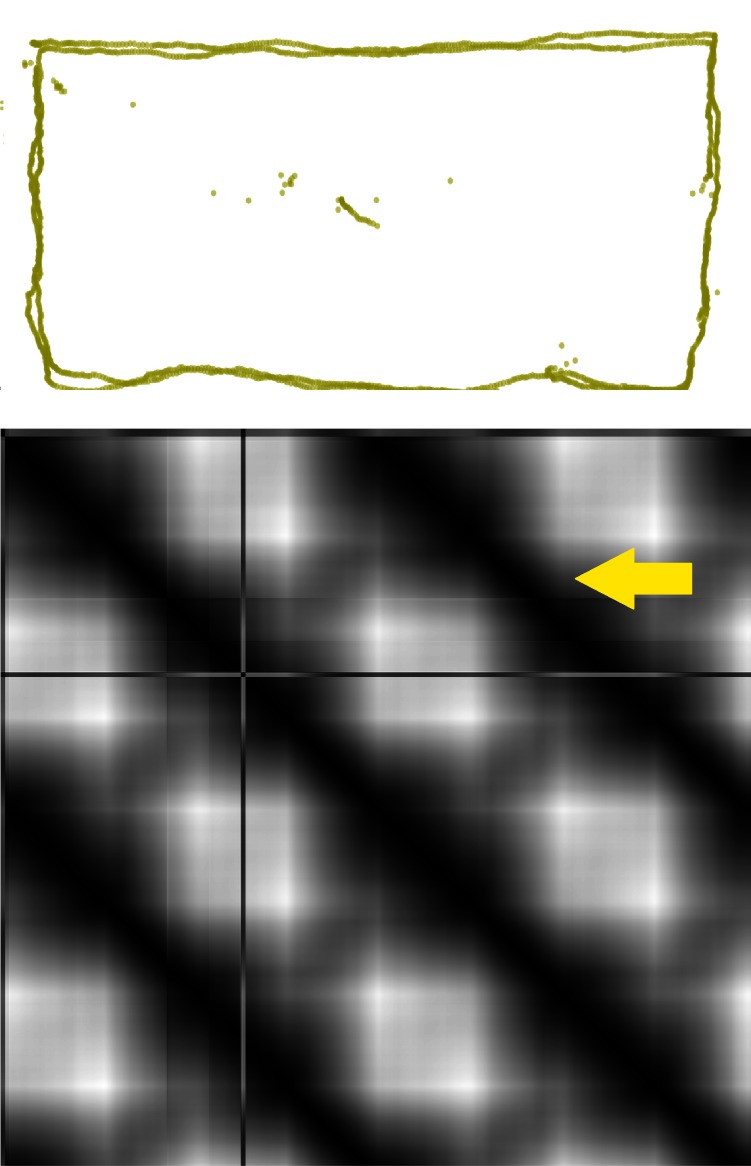
The GSSP showing smooth pursuit after the point wandering round the screen (scan-path visible above). The whole distance was covered twice. Black lines above and below diagonal represent recurrent recordings (yellow arrow points to one of the lines). The whitest points represent distances between gazes recorded in left-upper corner and right-bottom one. There are no squares with sharp edges, all edges are blurred.

Smooth pursuits are much slower eye movements than saccades, occurring when somebody is following with eyes a slowly moving object. Unfortunately, algorithms commonly used for the fixation detection frequently mistakenly classify smooth pursuits as fixations or saccades [ 11 ]. Smooth pursuits are also difficult to visualize. We conduced experiment showing that, based on the GSSP, it is easy to distinguish smooth pursuits and fixations, because edges of rectangles representing the former event are smoother (Figure 6).

### Distinguishing focal and ambient patterns

Two modes of processing visual information are commonly known: the focal and ambient processing [ 12 ] [ 13 ] which are used for the purpose of two different tasks: exploration and inspection. Short duration fixations followed by long saccades are characteristic for the ambient processing, while longer duration fixations followed by shorter saccades are indicative of the focal processing [ 14 ]. The visualization of eye movement that takes an ambient/focal processing into account is not a simple task. One of the attempts dealing with this issue may be found in [ 1 ], where ambient and focal fixations were distinguished by the usage of different coloring.

**Figure 7:**
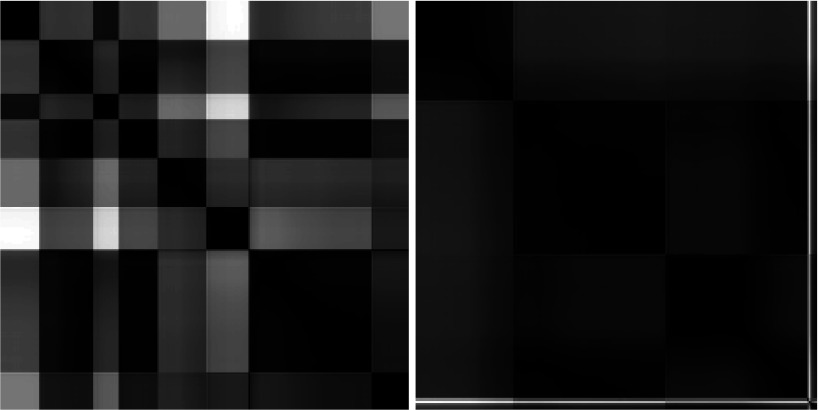
Two GSSPs showing the ambient (left) and the focal (right) processing of an image. There are many short fixations (small black squares along the diagonal) and long saccades (white rectangles adjacent to fixation squares) on the left plot while there are only few big black squares with short (dark) saccades on the right.

Assuming that the GSSP is a good tool for the ambient-focal distinction we have undertaken an appropriate experiment. Figure 7 shows two examples of plots. One of them is an example of ambient processing - a person is looking for something in the scene. Another one is a typical example of a focal processing - only some interesting objects are carefully inspected.

### Searching strategy

**Figure 8:**
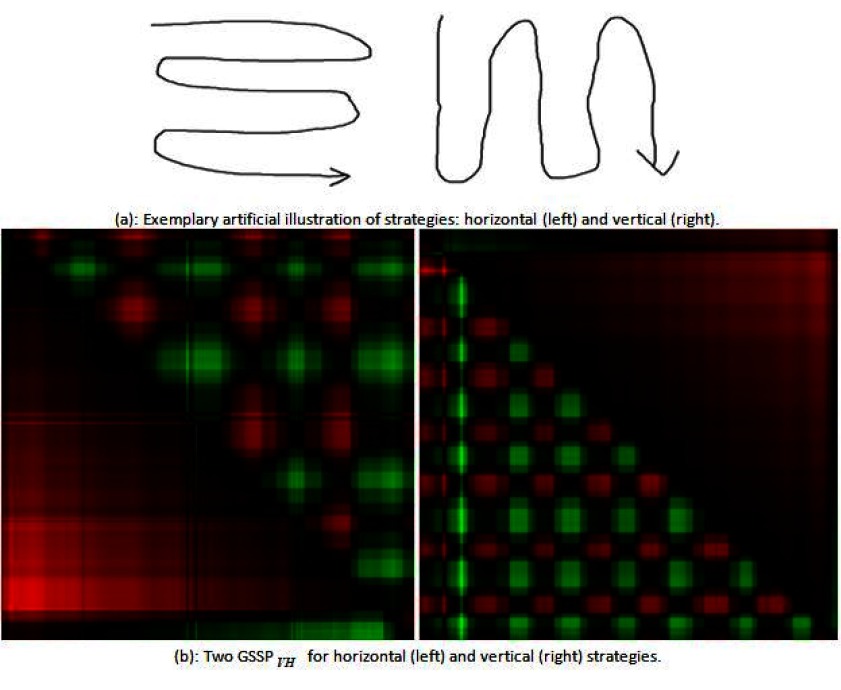
An example of GSSPs for different search strategies.

As the GSSP reveals both spatial and temporal patterns on one plot, it may be used to analyze strategies while exploring an image. Figure 8a presents two basic strategies: horizontal and vertical which are easily distinguishable in GSSP_VH_ shown in Figure 8b.

For the horizontal search strategy a person exploring the scene starts eye movement from the left upper corner and moves eyes to the right, thus all subsequent gaze positions are always to the right or in the same place (i.e. near the left edge of the scene). It is represented by red and black regions in the first row of the upper part of the plot. The whole horizontal (upper right) part of the GSSP_VH_ consists of subsequent red and green regions of similar size, which indicates that gaze was moving left and right with the similar speed. The part of the plot below the diagonal (visualizing vertical movements) is only black and red with very sparse green components, because vertical eye movements are made only downwards.

In the case of the vertical strategy similar color layout may be found, yet it is visible in lower and upper plot’s parts.

### Reading patterns

**Figure 9:**
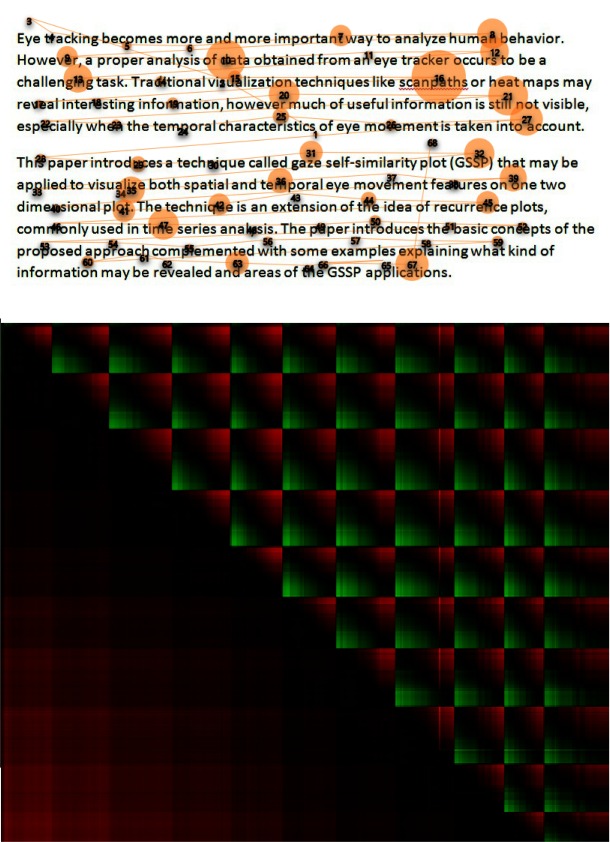
A scan-path (above) and the corresponding GSSP_VH_ (below) during reading of a text. It is visible that vertical movements are only directed downwards, while horizontal movements are slow to the right and very fast to the left.

Fixations’ patterns during reading are very specific, which makes the GSSP obtained for reading tasks also very specific. Analyzing the GSSP_VH_ presented in Figure 9 it may be noticed that vertical movements are only directed downwards, while horizontal ones are both to the left and to the right. Subsequent lines of text are easily distinguishable on the horizontal (upper right) part of the GSSP. It consists of squares with red upper right part and green lower left part which indicates that there were slow movements to the right and then rapid movements to the left (which makes it different from the GSSP for the horizontal search strategy presented in Figure 8b).

**Figure 10:**
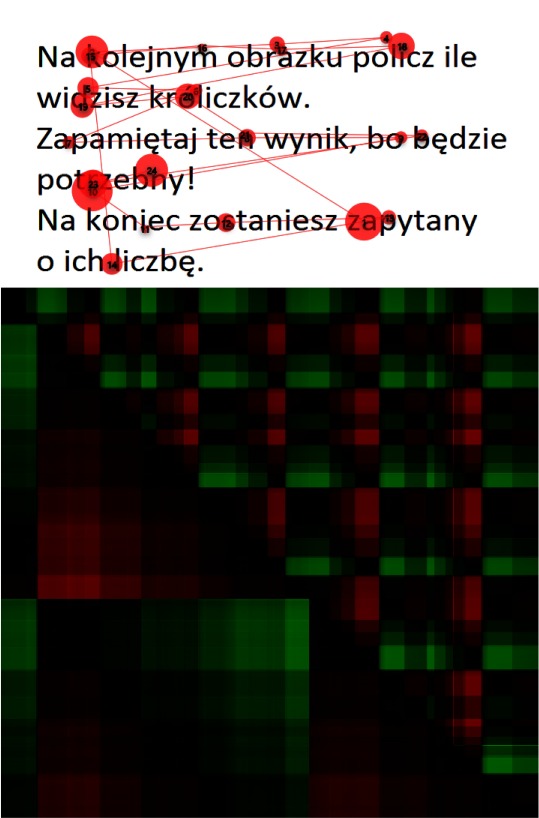
A scanpath during text reading (above) and the corresponding GSSP_VH_ (below). The vertical (lower left) part of the GSSP_VH_ reveals that the same sequence was read twice.

Another example of the text reading task is presented in Figure 10. A careful examination of the vertical (lower left) part of the GSSP_VH_ reveals that the same sequence repeats twice, which means that the same text was read twice. It is not so obvious when looking only at the scan-path.

**Figure 11:**
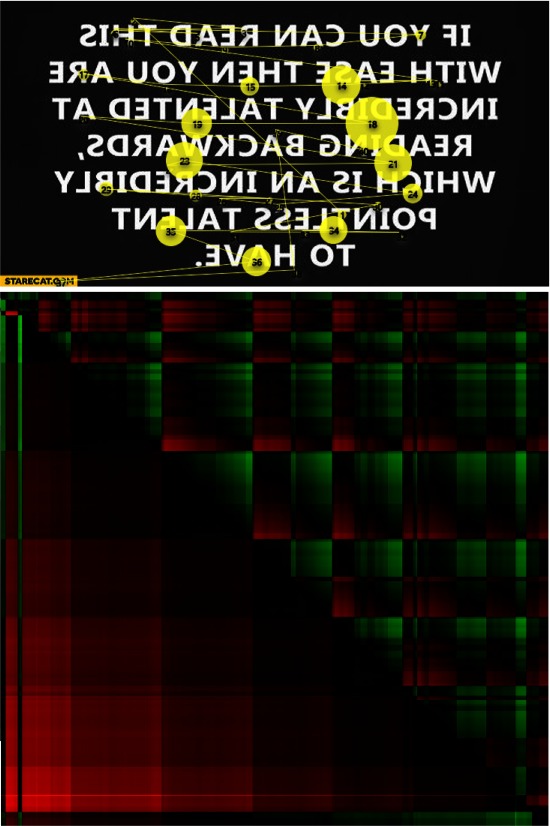
A scan-path during backward reading (above) and the GSSP_VH_ for this scan-path (below).

The subsequent example is a backward reading task. Figure 11 presents the scan-path and the GSSP_VH_ for such a task. It is visible that this time the horizontal part of the plot consists of rectangles with green upper right corner and red lower left corner which indicates slow movements to the left and rapid ones to the right. However, the pattern is not so clear, as in the case of the normal text reading, because the person was not used to this kind of reading.

**Figure 12:**
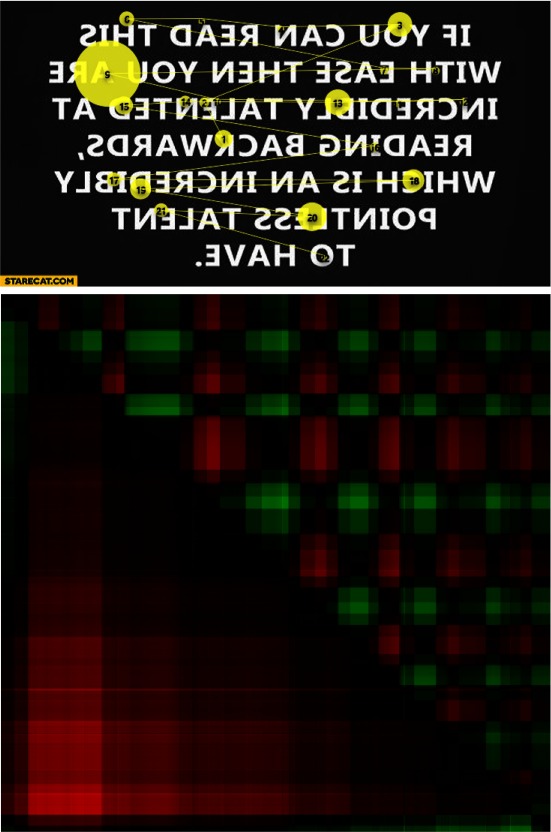
A scan-path during backward reading (above) and the GSSP_VH_ for this scan-path (below) for another person.

The same text was presented to another person and the corresponding scan-paths and GSSP_VH_ are presented in Figure 12. This time the person had serious problems with reading from right to left and it is visible on the GSSP_VH_ - the rectangles are not similar to the previous ones - movements to the right and to the left have similar velocity (such as in the case of the horizontal search strategy). It is worth noting that this information is not visible on scan-paths which look similarly in Figures 11 and 12.

### GSSP metrics usage

Our assumption was that the metrics presented in Method section (contrast, homogeneity and uniformity) may reveal interesting information about the gaze patterns. To check it, all three metrics for [0,1] offset were calculated for the first three seconds of five GSSPs presented in the previous sections (Table 1). This way we were able to compare metrics for normal and smooth pursuit observations (first row) and for ambient and focal observations (second row). The differences are visible for all compared observations, especially in the case of contrast and uniformity. The third row of the table shows comparison between the same metrics calculated for the same gaze pattern presented in Figure 9, but separately for horizontal and vertical directions.

**Table 1:** Metrics calculated for [0,1] offset for some GSSPs presented in the previous sections.

observation	contrast	homog	uniform
Normal (Fig. 5)	0.062	0.979	0.237
Smooth pursuit (Fig. 6)	0.034	0.987	0.430
Ambient (Fig. 7)	0.092	0.972	0.259
Focal (Fig. 7)	0.009	0.996	0.632
Text horizontal (Fig. 9)	0.082	0.959	0.370
Text vertical (Fig. 9)	0.011	0.994	0.664

### Distinguishing picture types

The next step in ascertaining the usefulness of the proposed metrics was utilizing them in distinguishing visual behavior depending on an image type.

**Figure 13:**
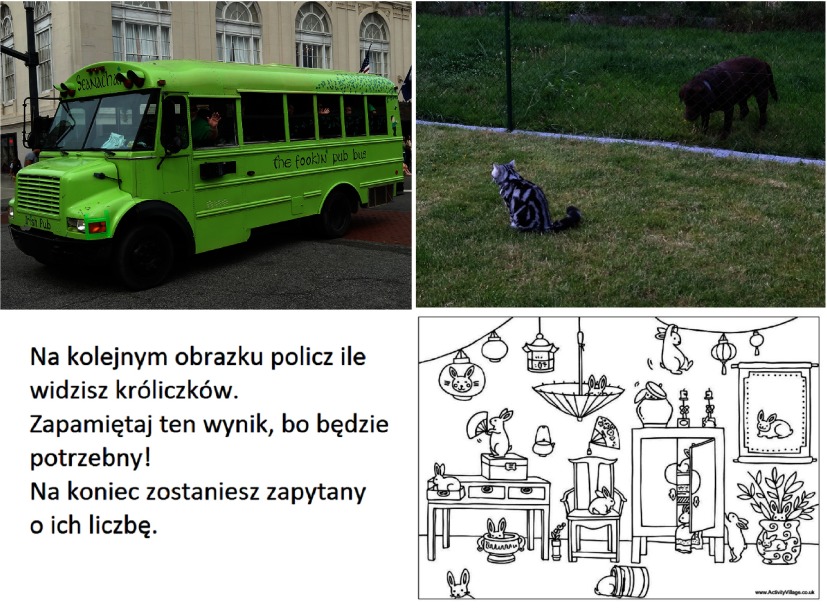
Four images analyzed during the first experiment.

The dataset used for this purpose consisted of gaze recordings registered for 18 participants looking at four images - two free observation images denoted as ’bus’ and ’cat’, one image with text to be read (’text’) and one image for which the participants’ task was to count the number of rabbits. All four images are presented in Figure 13. After removing two bad samples the remaining subset formed a dataset consisting of 232 observations.

For each of them the GSSP_VH_ was created and three metrics - contrast, homogeneity and uniformity - calculated separately (1) for every direction (horizontal - upper right triangle and vertical - lower left triangle) and (2) for three different offsets: (0,1), (1,0) and (1,1). It gave overall 18 attributes derived from one GSSP corresponding to one observation.

During the metrics analysis it occurred that values of uniformity calculated for the same direction (V or H) and for different offsets are highly correlated (Pearson correlation for every pair >.9). Therefore, it was decided to remove those metrics determined for (0,1) and (1,0) offsets from the further studies. After that step there were 14 attributes describing every GSSP_VH_ (and this way every observation).

**Figure 14:**
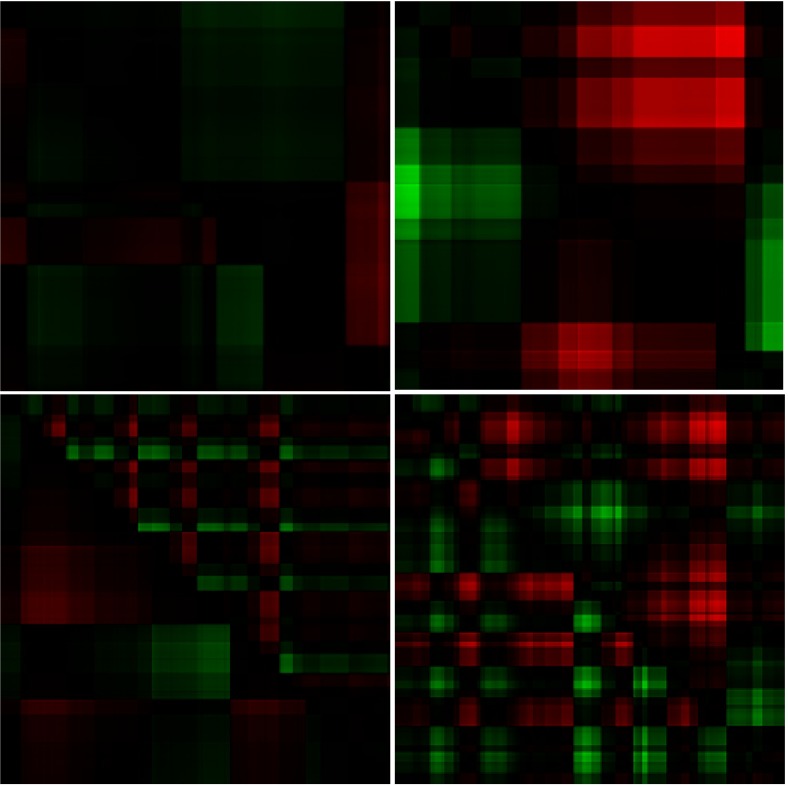
The GSSPs of one observer for four images presented in Figure 13. The order is the same as in Figure 13.

**Figure 15:**
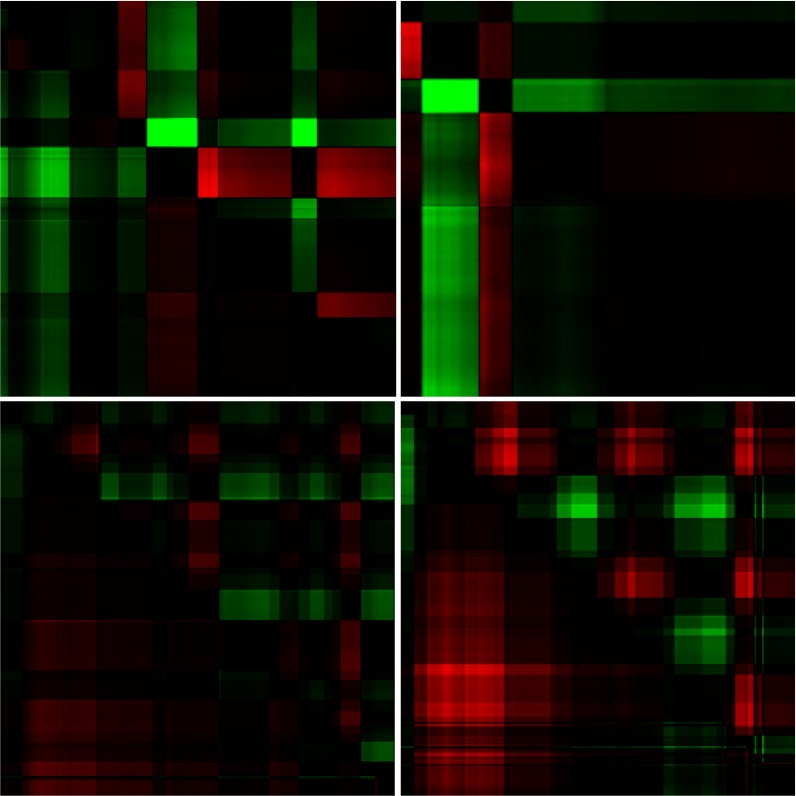
The GSSPs of another observer for four images presented in Figure 13. The order is the same as in Figure 13

The resulting GSSPs for all four images and two exemplary participants are presented in Figures 14 and 15.

The mean values of attributes calculated for each image are presented in Table 2. Because, according to Shapiro-Wilk test, none of the 14 analyzed attributes exhibited normal distribution, the nonparametric Kruskal-Wallis test was utilized to check, if there are differences in attributes values among images. The differences were significant, so the post-hoc pairwise comparison realized by means of Mann-Whitney test was also calculated (see Table 3).

**Table 2:** Mean attribute values for different images averaged for all 18 participants. Standard deviation in brackets. Kruskal-Wallis test result in column H and significance in column p-value

attribute	bus	cat	text	task	H	p-value	sign
H01 contrast	.062 (.048)	.083 (.042)	.154 (.064)	.063 (.037)	28.451	0	***
H01 homogeneity	.977 (.01)	.969 (.011)	.952 (.007)	.975 (.006)	34.253	0	***
H10 contrast	.069 (.048)	.079 (.067)	.141 (.075)	.058 (.025)	22.321	0	***
H10 homogeneity	.977 (.01)	.973 (.013)	.957 (.01)	.978 (.006)	32.848	0	***
H11 contrast	.125 (.086)	.154 (.092)	.286 (.133)	.114 (.052)	25.403	0	***
H11 homogeneity	.957 (.017)	.947 (.02)	.917 (.013)	.957 (.01)	35.417	0	***
H11 uniformity	.25 (.125)	.205 (.056)	.152 (.023)	.159 (.024)	23.627	0	***
V01 contrast	.045 (.016)	.051 (.022)	.074 (.022)	.07 (.015)	21.51	0	***
V01 homogeneity	.979 (.007)	.977 (.009)	.973 (.006)	.972 (.005)	12.853	0.005	**
V10 contrast	.063 (.024)	.056 (.019)	.074 (.028)	.075 (.019)	10.251	0.017	*
V10 homogeneity	.976 (.007)	.975 (.007)	.97 (.01)	.97 (.005)	10.218	0.017	*
V11 contrast	.098 (.034)	.097 (.035)	.136 (.043)	.135 (.03)	16.655	0.001	***
V11 homogeneity	.96 (.011)	.957 (.013)	.949 (.013)	.948 (.008)	13.832	0.003	**
V11 uniformity	.363 (.115)	.311 (.111)	.205 (.048)	.156 (.023)	47.07	0	***

**Table 3:** The results of Mann-Whitney test for significance of differences between each pair of images averaged for all 232 observations (and 18 participants). The table shows p-values for each attribute and pair. ‘*’ means p-value<0.01, ‘**’ - p-value<0.001.

	bus-cat	bus-text	bus-task	cat-text	text-task	cat-task
H01 contrast	.09	**	.24	**	**	.25
H01 homog	.04	**	.21	**	**	.22
H10 contrast	.42	**	1.0	**	**	.19
H10 homog	.29	**	.66	**	**	.14
H11 contrast	.19	**	.66	**	**	.14
H11 homog	.06	**	.62	**	**	.08
H11 uniformity	.19	**	**	**	.48	*
V01 contrast	.41	**	**	*	.79	*
V01 homog	.58	*	*	.04	.47	.01
V10 contrast	.32	.24	.08	.03	.82	*
V10 homog	.82	.04	*	.1	.91	.01
V11 contrast	.96	*	*	*	.76	*
V11 homog	.64	.01	*	.06	.81	*
V11 uniformity	.08	**	**	**	**	**

The above-presented results with their statistically significant differences showed that distinguishing image types based on calculated GSSP metrics is possible. To confirm the findings a subsequent step of the analysis was undertaken, in which all 14 attributes were used to associate an observation with an image type. That classification process was performed by means of Random Forest classifier with one leave out cross validation using WEKA implementation with default parameters [ 15 ]. The resulting confusion matrix is presented in Table 4. It is visible that ’text’ and ’task’ were the easiest images to classify (17 out of 18 and 16 out of 18 correct classifications, respectively). On the other hand the ’bus’ and ’cat’ images both representing the free viewing visual pattern - were frequently mistaken with each other.

**Table 4:** Confusion matrix for the images classification. Each cell shows how many instances of the actual class defined in the column were classified as the class defined in the row.

actual -> predicted	bus	cat	text	task
bus	11	5	0	1
cat	5	10	1	0
text	1	3	17	1
task	1	0	0	16

### Distinguishing a level of expertise

One of the intensively studied issues regarding utilizing eye tracking methods is revealing eye movement patterns of people with various levels of expertise, which is especially visible in medicine. For this reason the next test aimed to check, if the GSSP may be used to distinguish gaze patterns for laymen and specialists. The dataset utilized in the analysis consisted of eye movement recordings of 8 laymen and 8 specialists looking at 12 X-rays for 5 seconds (the duration was chosen arbitrarily). The set of images included chest X-rays with and without various diseases. Participants’ task was to explore each image and assess it based on four possibilities provided.

Similarly to the previously described case, there was the GSSP_VH_ created for every observation and three attributes: contrast, homogeneity and uniformity calculated separately for both directions and three different offsets.

In this case it occurred that values of all attributes calculated for the same direction (V or H) and for different offsets are highly correlated (Pearson correlation for every pair >.8). Therefore, only (1,1) offset was taken into account. The mean values of attributes for each group together with Kruskal-Wallis test results are presented in Table 5.

**Table 5:** Mean attribute values for all 216 observations (12 images and 18 participants) with standard deviation in brackets. Kruskal-Wallis test results are provided in column H and significance in column p-value

attribute	laymen	specialists	H	p-value
H cont	.14 (.16)	.09 (.06)	0.13	0.72
H homo	.94 (.05)	.96 (.02)	0.2	0.66
H unif	.4 (.12)	.29 (.07)	43.8	0
V cont	.2 (.21)	.11 (.07)	14.4	0
V homo	.93 (.06)	.96 (.02)	13.0	0
V unif	.32 (.15)	.25 (.09)	9.5	0.002

**Figure 16:**
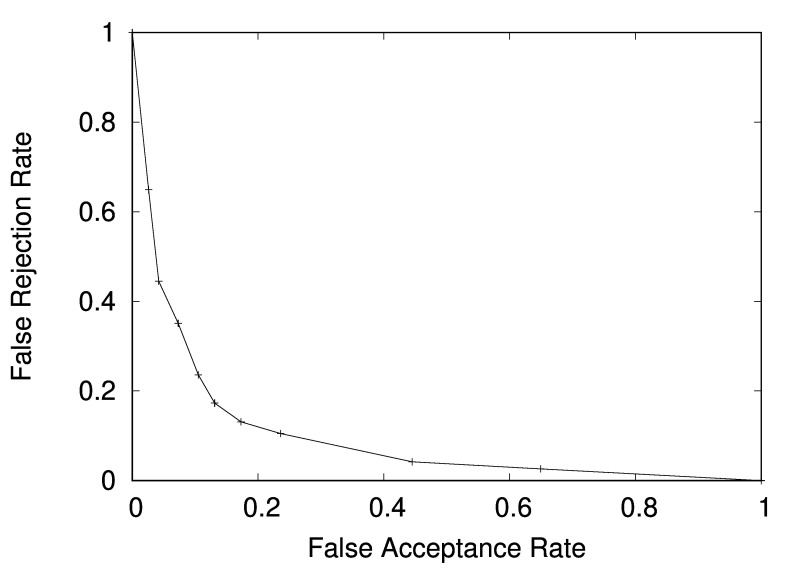
DET curve for the specialist-layman prediction based on calculated GSSP metrics.

Similarly to the previous experiment, all six attributes of each observation were used to classify it as specialist’s or layman’s. Once more Random Forest classification algorithm using WEKA implementation with default parameters [ 15 ] was applied. The accuracy of the classification was 85% and the confusion matrix is presented in Table 6. Additionally the Detection Error Tradeoff (DET) curve for specialist-layman prediction is presented in Figure 16.

**Table 6:** Confusion matrix for the experts’ classification. Each cell shows how many instances of the actual class defined in the column were classified as the class defined in the row.

actual -> classified as	laymen	specialists
laymen	83	17
specialists	12	79

Moreover, when the classification results of the same person were summarized for 12 images observations with the usage of a classic voting algorithm - all participants were classified correctly either as a layman or specialist (8 out of 8 correct for both classes). Such results may be treated as the confirmation of the GSSP usefulness for distinguishing laymen and specialists.

### Handling with long sequences

**Figure 17:**
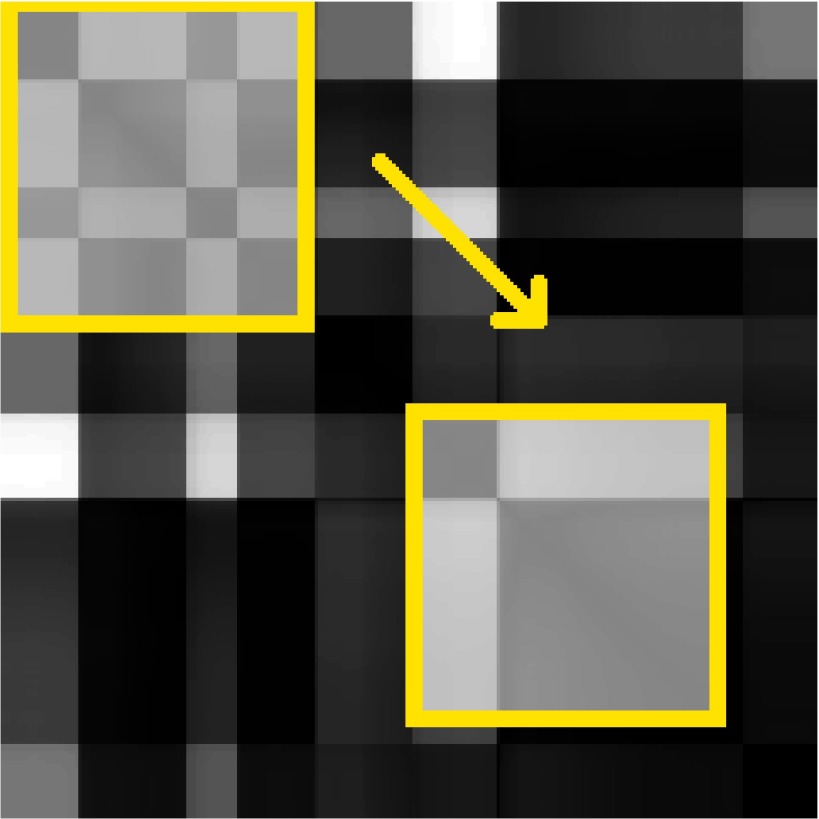
Calculation of the GSSP in a moving window.

Visualization techniques very often have to deal with problems of large amount of samples. Presenting big numbers of fixations and saccades makes scan-paths or heat maps difficult to analyze, especially in regard to detailed information. The problem may be overcome by analyzing data taking its smaller parts into account. Similar solution may be used in the case of the GSSP. If a gaze sequence (scan-path) is very long (e.g. during watching a movie) it is not necessary to analyze the whole GSSP - the better option is to create multiple GSSPs for successive periods. The idea is visually presented in Figure 17, where parts were selected from the whole GSSP . Such extracted GSSPs may be then compared to find characteristic moments during observation.

**Figure 18:**
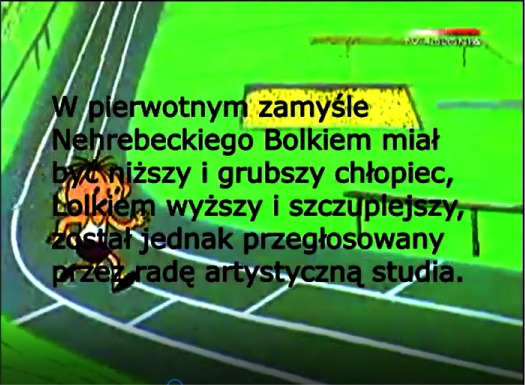
One frame from the cartoon movie with a text displayed.

That GSSP feature was investigated during the next experiment aimed to check, if it was possible to find out, based on GSSP metrics, if a person was reading a text. For the sake of the experiment a cartoon movie was used. From time to time a foreground text appeared on a screen (see Figure 18).

There were six various texts displayed during the movie with different durations from 7 to 9 seconds and short breaks (2-5 seconds) between subsequent texts presentations. A participant’s task was to watch the movie, but at the same time to read all texts.

The research question was to ascertain, if it was possible to indicate whether a person was reading the text while watching the movie, based on metrics values. To answer this question at first the GSSPs were created for one-second windows with 0.16 second step. Then, all metrics were calculated separately for each GSSP and their values were ascertained in terms of the ’text visibility’-’metrics values’ correlation existence. For this purpose the function defining moments of text presentation was defined as:

(15) $$\begin{array}{ccc} \; & textvisible\left(t\right)=\left\{ \begin{array}{cc} 1, & text\;visible\\ 0, & text\;not\;visible \end{array}\right. & \; \end{array}$$

where textvisible(t) indicates whether in time t a text was visible on the screen (the function value is 1) or not (function value is 0).

**Figure 19:**
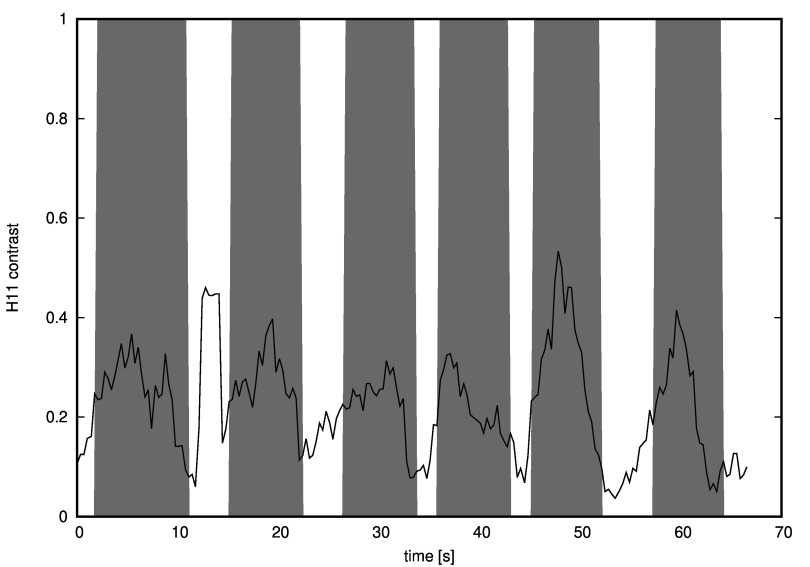
Horizontal contrast values calculated in a moving window of approximately 1 second. Grey areas are moments when a text was displayed as a foreground. The correlation is clearly visible - the only exception is a moment between the first and the second text appearance, when the contrast is higher than expected.

**Figure 20:**
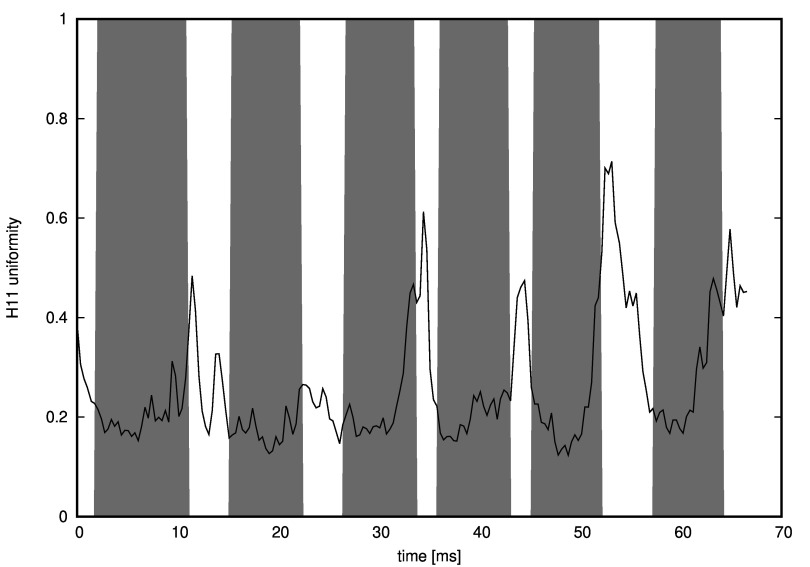
Horizontal uniformity values calculated in a moving window of approximately 1 second. Grey areas are moments when a text was displayed as a foreground. The uniformity is clearly lower when there is the text to be read.

It occurred that Pearson correlation between horizontal contrast and the outcome of textvisible(t) function was 0.46 (see Figure 19) and between horizontal uniformity and the textvisible(t) function values was -0.54 (see Figure 20).

When a participant’s task was defined as: ’watch the movie and do not pay attention to texts’ there was no correlation between metrics and textvisible(t) function results found.

## Discussion

Experiments presented in the previous section showed that the GSSP may be used as a useful tool in various fields of eye movement analysis. It may be utilized to check the quality of recordings by a convenient outliers presentation. Additionally, different plot patterns provided for different tasks makes the GSSP helpful in identifying underlying activity such as smooth pursuit, reading or searching task. Moreover, the GSSP ensures the possibility of recognizing the way a scene is observed - a direction of the scene scanning and ambient/focal characteristic of its exploration.

However, the GSSP is not only a visual tool for gaze pattern analysis, but it may also be used to calculate meaningful and quantitative metrics, which may enrich our understanding of eye movements. For instance when we compare metrics for GSSP presented in Figure 5 and metrics of smooth pursuit GSSP (Figure 6), it is visible that contrast is much lower for the latter, while uniformity is higher (see Table 1).

When ambient and focal GSSPs (Figure 7) are compared, the GSSP for focal observation is characterized with much lower contrast, slightly higher homogeneity and much higher uniformity (Table 1).

Usage of GSSP_VH_ offers opportunity to compare metrics obtained for horizontal and vertical directions. When these metrics are compared for text reading GSSP (Figure 9) it occurs that the contrast is lower and both homogeneity and uniformity are higher for vertical direction (Table 1).

### Distinguishing picture types

The results presented in Table 2 revealed significant effect among images for all attributes derived from GSSP. The differences were especially visible for attributes calculated for horizontal part of the GSSP.

The post-hoc pairwise comparison realized by means of Mann-Whitney test revealed that there were no significant differences between ’bus’ and ’cat’ observations (Table 3). However, all horizontal attributes values showed significant differences when comparing both free observations (’cat’ and ’bus’) with ’text’ one. On the other hand, there were no significant differences for horizontal contrast and homogeneity between free observations and ’task’ explorations, but there were some significant differences for vertical attributes. Horizontal contrast and homogeneity as well as vertical uniformity significantly distinguishes ’text’ and ’task’ observations.

The classification results presented in Table 4 show that it was possible to differentiate observation based on its purpose. The ’text’ and ’task’ observations were classified correctly with accuracies 88% and 94% respectively while ’cat’ and ’bus’ observations were frequently misclassified.

The careful analysis of the results leads to the following conclusions:

• ’text’ has significantly higher horizontal contrast and lower horizontal homogeneity than other images,

• ’task’ has significantly lower horizontal contrast than other images,

• both ’task’ and ’test’ have significantly lower uniformity and higher vertical contrast than both ’free observation’ images,

• ’free observation’ images have similar attributes values and no significant differences between them were observed,

• it is possible to distinguish the type of observation taking into account only three metrics derived from the GSSP, which was demonstrated using the Random Forest classification algorithm.

### Distinguishing a level of expertise

The results presented in Table 5 reveal that the uniformity and vertical contrast are significantly lower for specialists whereas the vertical homogeneity is higher. It suggests that specialists’ gaze pattern is more sophisticated - there are different jumps/saccades to different directions and there are no dominant directions, which results in lower uniformity. At the same time the jumps/saccades (especially in vertical direction) are shorter, which results in lower contrast and higher homogeneity. Additionally, the standard deviations of metrics among specialists are lower than among laymen.

Based on those outcomes it may be concluded that specialists observe the image more carefully - they focus their attention on relevant parts of the image (more or less the same for each specialist), whereas laymen just scan the image - using similar and predictable patterns for each image (but specific to each observer).

The classification part of the experiment showed that it is possible to distinguish a layman and a specialist gaze patterns taking into account only three metrics derived from the GSSP. With 12 gaze patterns available for a person the classification algorithm performed perfectly in predicting the person’s level of expertise.

### Handling with long sequences

The last (movie) experiment described in the previous section leads to the conclusion that the proposed technique is scalable towards long sequences of recordings. By dividing them into shorter series with the application of arbitrarily defined windows, within - as well as between - series comparison is facilitated. Additionally, the results obtained showed the usefulness of the proposed metrics, with the example of the horizontal contrast and horizontal uniformity metrics, which may be good indicators, if a person is reading a text.

## Summary

The eye movement analysis attracts interest of scientists from many fields of research and it has become a promising tool for the exploration of human brain functioning [ 16 ]. The aim of the paper was to present the new method for eye movement visualization, which would be capable to overcome the limitation present in most other solutions, i.e. the difficulty in simultaneous presentation of spatial and temporal eye movement characteristics.

The developed method - The Gaze Self-Similarity Plot (GSSP), based on recurrence plot technique - achieves it by means of a single two-dimensional plot. The most important features of this solution are the usage of raw gaze points instead of fixations and encoding distances between gazes as continuous values. Both features make the GSSP completely independent of any thresholds or initial assumptions. By introducing its extended version - the GSSP_VH_ encoding horizontal and vertical movements in different ways and using colors to distinguish the sense of the movement, more information is available on the same plot.

 Along with the method description, the discussion of its possible applications was also provided. Among them effortless revealing reading patterns, outliers, ambient/focal characteristics or differentiating search strategies may be mentioned. The presented solution was equipped with several metrics as well. They allow for both quantitative GSSP’s assessment and comparison of various such plots. Two examples of their usage were discussed in the paper: (1) for distinguishing picture types and (2) for distinguishing levels of expertise. In both cases statistical analysis revealed significant differences in metrics values for studied groups. These findings were confirmed by results obtained during the classification process performed to assign an observation to one of these groups.

Furthermore, based on eye movements gathered while watching a cartoon movie with overlapping text, the example of processing gaze sets consisting of big amount of recordings was provided. The example also showed that by means of the GSSP it is feasible to detect which of the elements overlapping on the screen - movie or text - attracted user’s attention. This distinction is hard to achieve when using other visualization techniques.

All the presented GSSP’s applications give - in authors’ opinion - strong evidence that the GSSP may be a valuable supplement to other, existing gaze pattern visualization techniques. It should also be emphasized that the list is not exhaustive and many other measures, metrics and interpretations may be taken into account - those issues may constitute a basis of a future analysis.

### Acknowledgements

The research presented in this paper was partially supported by the Silesian University of Technology grant BK/263/RAu2/2016. The authors declare that no conflicts of interest exist.
